# Carbon Nanotube-Based Thermoelectric Modules Enhanced by ZnO Nanowires

**DOI:** 10.3390/ma15051924

**Published:** 2022-03-04

**Authors:** Patrycja Taborowska, Tomasz Wasiak, Mika Sahlman, Mari Lundström, Dawid Janas

**Affiliations:** 1 Department of Organic Chemistry, Bioorganic Chemistry and Biotechnology, Silesian University of Technology, B. Krzywoustego 4, 44-100 Gliwice, Poland; patrycja.taborowska@polsl.pl (P.T.); tomasz.wasiak@polsl.pl (T.W.); 2 Hydrometallurgy and Corrosion, Department of Chemical and Metallurgical Engineering (CMET), School of Chemical Engineering, Aalto University, P.O. Box 16200, FI-00076 Aalto, Finland; mika.sahlman@aalto.fi (M.S.); mari.lundstrom@aalto.fi (M.L.)

**Keywords:** carbon nanotubes, ZnO nanowires, thermoelectric properties

## Abstract

Carbon nanotubes (CNTs) have a wide range of unique properties, which have kept them at the forefront of research in recent decades. Due to their electrical and thermal characteristics, they are often evaluated as key components of thermogenerators. One can create thermogenerators exclusively from CNTs, without any metal counterpart, by properly selecting dopants to obtain n- and p-doped CNTs. However, the performance of CNT thermogenerators remains insufficient to reach wide commercial implementation. This study shows that molecular doping and the inclusion of ZnO nanowires (NWs) can greatly increase their application potential. Moreover, prototype modules, based on single-walled CNTs (SWCNTs), ZnO NWs, polyethyleneimine, and triazole, reveal notable capabilities for generating electrical energy, while ensuring fully scalable performance. Upon doping and the addition of ZnO nanowires, the electrical conductivity of pure SWCNTs (211 S/cm) was increased by a factor of three. Moreover, the proposed strategy enhanced the Power Factor values from 18.99 (unmodified SWCNTs) to 34.9 and 42.91 µW/m∙K^2^ for CNTs triazole and polyethyleneimine + ZnO NWs inclusion, respectively.

## 1. Introduction

The progressing electrification of our daily lives puts more and more pressure on the electric grid [1,2,3]. A modern house contains numerous appliances, each of which requires electrical energy. Furthermore, various gadgets, such as smartphones, smartwatches, etc., used by us also require such resources to operate. Therefore, more power plants are needed to fulfil the future demand. However, electrical energy generation is currently inefficient, and more than half of the energy is lost in the process as waste heat [4]. It is, therefore, highly reasonable to address the challenge of the low overall yield of the current approach.

New types of materials, which emerged at the turn of the XX and XXI centuries, may solve this problem. In particular, carbon nanostructures, such as carbon nanotubes (CNTs) [5] have highly useful characteristics, making them attractive in this area. They may provide remarkable electrical [6,7] and thermal [8,9] properties, and even outperform copper, in terms of current carrying capacity [10,11] and thermal conductivity [12]. Among the most important attributes of CNTs is the capability to induce thermoelectric voltage when subjected to a temperature gradient [13,14]. The thermoelectric capability of CNTs is quantified by a parameter referred to as the Seebeck coefficient, which quantifies the amount of electrical charge generated per unit temperature.

As highlighted in a comprehensive review on the topic by Blackburn and colleagues [15], CNTs are well suited for harvesting low-grade heat in the 300–400 K temperature regime. They can provide an alternative to typical inorganic thermoelectric materials, designed for operation above 600 K (e.g., Bi_2_Te_3_, PbTe), which are toxic, rigid and based on metals of limited abundance [16,17,18]. In contrast, thermogenerators based on CNTs are flexible, and the constituting CNTs can be made from a myriad of carbon-containing renewable chemical compounds [19]. Furthermore, they have highly tunable characteristics [20,21], which can be tailored by exercising an appropriate modification strategy. One of the key attributes of CNTs is that they are amphoteric, which means that they can be both p- or n-doped [22]. Consequently, p-type and n-type thermoelements, constituting thermoelectric modules, can be made using CNTs. CNTs are naturally p-doped in the ambient environment [23,24], so researchers’ current focus is on finding chemical compounds or materials that can make high-performance n-type thermoelements from CNTs. Various electron-rich molecular dopants, such as 1,3-Bis (diphenyl-phosphino) propane, poly (4-vinylpyridine) or Tetronic 1107, have been tested [22]. Chemical compounds, however, may be prone to desorption or degradation (especially at high temperature).

In this work, we investigate the possibility of using ZnO nanowires (NWs), having n-type characteristics [25], to enhance the thermoelectric performance of modules based on single-walled CNTs (SWCNTs). It is essential that they are not made of critical raw materials like those mentioned above. Pure SWCNTs were n- and p-doped and the effect of ZnO NW addition on the values of electrical/thermal conductivity, Seebeck coefficient, Power Factor, and Figure of Merit (zT) was analyzed. The study demonstrates that the addition of ZnO NWs is highly beneficial for the SWCNT-based thermoelectric modules.

## 2. Materials and Methods

### 2.1. Chemical Compounds

SWCNTs (Tuball™, OCSiAl, Leudelange, Luxemburg), zinc nitrate hexahydrate (Acros Organics, Geel, Belgium, 98%), sodium hydroxide (Chempur, Piekary Slaskie, Poland, pure p.a.), sodium dodecyl sulfate (Sigma-Aldrich, Saint-Louis, MO, USA; 92.5–100.5%), acetone (Avantor, Gliwice, Poland), toluene (Avantor, Gliwice, Poland), were obtained from the indicated vendors.

### 2.2. Synthesis of ZnO NWs

First, 20 mL of 1 M Zn(NO_3_)_2_ water solution was poured into a (250 mL) round-bottom flask. The solution was diluted with 45 mL of distilled water and cooled down to 1 °C in an ice bath with salt. The mixture was continuously stirred, and then 30 mL of 4 M NaOH was added dropwise afterward. At first, a white precipitate could be observed, which slowly dissolved with each drop of the base. Then 5 mL of 0.2 M SDS solution was injected. The solution was stirred at room temperature for 1.5 h to obtain a white precipitate. Next, the flask was equipped with a condenser and placed in an oil bath heated up to 85 °C. The growth of ZnO NWs was conducted without stirring. The obtained product was filtered off, washed with copious amounts of distilled water, and dried in air.

### 2.3. Assembly of Doped SWCNT Films

**Figure 1:**
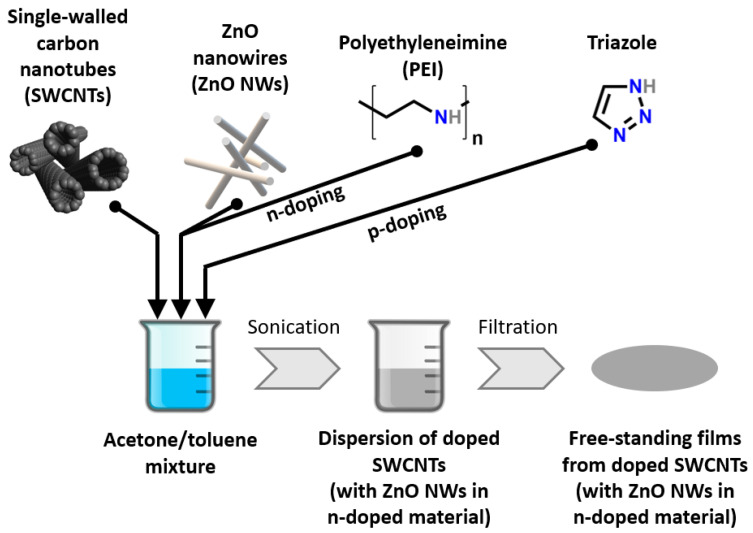
Assembly and doping of SWCNT films for thermoelectric applications.

SWCNTs were transformed into thin free-standing films by adapting a method reported by us previously [26]. The process is summarized in Figure 1. To make n- and p-doped SWCNT films, polyethyleneimine (PEI) or triazole were introduced to 80 mL of acetone/toluene mixture (1:1, *w*/*w*) at the concentration of 0.1 M, respectively. Then, SWCNTs and ZnO NWs (in the case of n-doping solution) were added in the amounts indicated in Table 1. Then, the mixture was sonicated over an ice bath until obtaining a uniform dispersion (UP200St sonicator, Hielscher, Teltow, Germany). Subsequently, it was filtered under reduced pressure onto membranes from Teflon (pore size: 0.45 µm, diameter: 47 mm; Fisherbrand, Ottawa, ON, Canada) using a Büchner funnel and a vacuum flask. The films formed on the filters were then kept in a desiccator overnight to facilitate the evaporation of the solvents. Once dried, they were delaminated from the support due to their low adhesion to PTFE. Thus, 2 mm × 40 mm specimens (thickness~100 µm) were produced from the films for analysis.

### 2.4. Characterization

Raman spectroscopy was employed to investigate the possibility of electronic and structural differences between the specimens. The inVia Renishaw Raman microscope (Renishaw, Wotton-under-Edge, UK) with a laser wavelength of λ = 514 nm acquired data from 10 to 3200 cm^−1^. Spectra were obtained at several sample areas using extended acquisition time to ensure statistical significance and high signal-to-noise ratio, respectively.

X-ray diffraction (XRD, Rigaku D-max 2500, Tokyo, Japan) was used to study the crystallinity of ZnO NWs. A CuKα monochromatic X-ray source was engaged to collect the data for 2θ between 30° and 80°. A 0.03° 2θ step size was set to resolve the recorded patterns properly.

Scanning Electron Microscope (SEM; Quanta 250 FEG, Hillsboro, OR, USA) was employed to investigate the microstructure of the material. Image acquisition was conducted at the acceleration voltage of 10 kV. The samples were not sputtered.

A custom made 4-probe setup was used to evaluate electrical conductivity of the material (Keithley 2450 SourceMeter, Cleveland, OH, USA). The specimens were attached to a sample holder using Ag conductive paint to avoid contact resistance. Resistance of the samples was then measured by supplying a 100 mA current, which did not elevate the temperature of the samples. The recorded values of resistance were recalculated into conductivity by considering the sample dimensions. Thickness was established using a micrometer screw gauge (Electronic Universal IP54, Linear Tools, Dunstable, UK).

A steady-state method with infrared thermography [27,28,29] was implemented to obtain thermal conductivity values analogously as in our previous work [30]. In brief, the temperature profile of the sample was analyzed in an evacuated chamber using a thermal camera (FLIR ETS 320, Wilsonville, OR, USA). The recorded data were then modeled using the following equation

(1) $$\kappa =\frac{U\cdot I\cdot 0.5L}{4\cdot w\cdot t\cdot \left(T_{m}-T_{0}\right)}$$

where *κ* is thermal conductivity, *U* is voltage, *I* is current, *L*, *w*, and *t* are length, width, and thickness of the sample, respectively. *T*_0_ and *T_m_* are temperatures of the sample ends and middle part, respectively.

Seebeck coefficient was determined from room temperature to 110 °C (SeebCam, LBR, Lublin, Poland) at the temperature gradient of 5 °C between the sample ends. The values of electric potential were registered under these conditions (the material was studied in an evacuated chamber to eliminate the influence of convection). The performance of modules containing 5, 10, and 15 n-p pairs was analyzed by heating one side of the module with a hot plate (temperature was verified with a Type K thermocouple). Open Circuit Voltage was measured across the above-mentioned temperature range with a source meter (Keithley 2450 SourceMeter, Cleveland, OH, USA) to confirm the utility of the developed solution.

The values of Power Factor (*PF*) and Figure of Merit (*zT*) were calculated using the following formula.

(2) $$PF=\sigma \cdot S^{2}$$

(3) $$zT=\frac{\sigma \cdot S^{2}}{\kappa }T$$

where *σ* is electrical conductivity, and *S* is Seebeck coefficient.

## 3. Results

**Figure 2:**
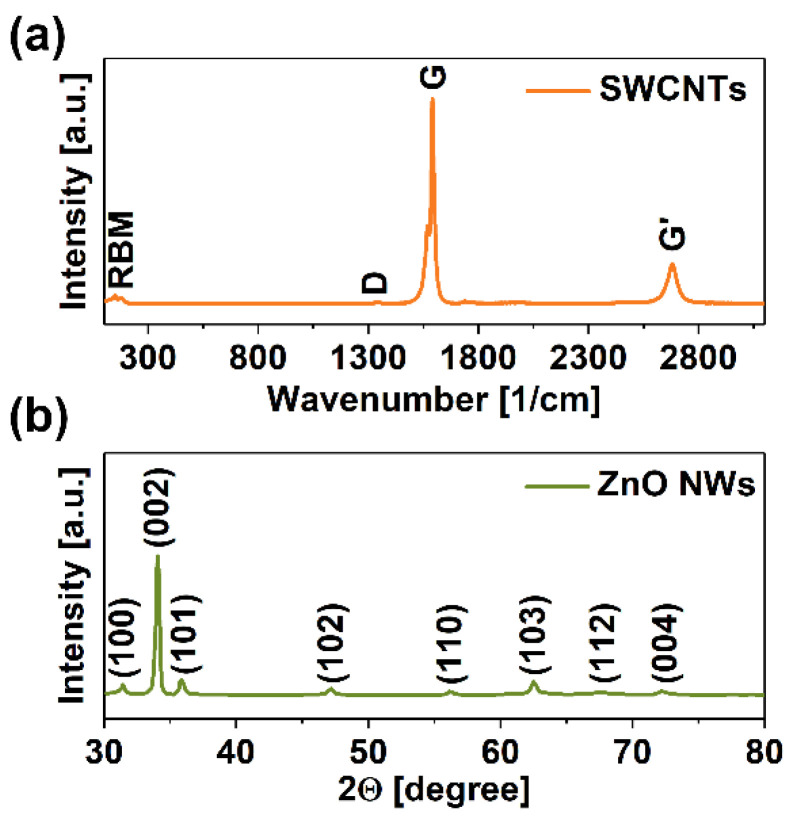
(**a**) Raman spectrum of SWCNTs and (**b**) XRD patterns of ZnO NWs used in the study.

First, the crystallinity of base materials, SWCNTs and ZnO NWs, was determined by Raman spectroscopy and XRD, respectively. The nanocarbon component was of high quality, as revealed by the negligible intensity of the defect-induced band D (Figure 2a). It typically corresponds to lattice defects, functional groups on the surface of SWCNTs, and the presence of other sp^3^-rich types of carbon [31]. On the other hand, the G peak, indicative of the vibration of carbon atoms of sp^2^ hybridization, was narrow and of high intensity. The splitting of this feature into G− and G+ components [32] and the emergence of the Radial Breathing Mode (RBM) confirmed that the CNTs were indeed single-walled. 

The ZnO NWs synthesized in-house also manifested a high degree of structural perfection (Figure 2b). The patterns were sharply defined and stayed in accordance with the features expected from a ZnO material. Zhou et al. [33] and Zappa et al. [25] previously reported matching XRD patterns for ZnO NWs. The obtained results mean that the NWs are exclusively made of high purity ZnO because of the absence of any other reflections.

**Figure 3:**
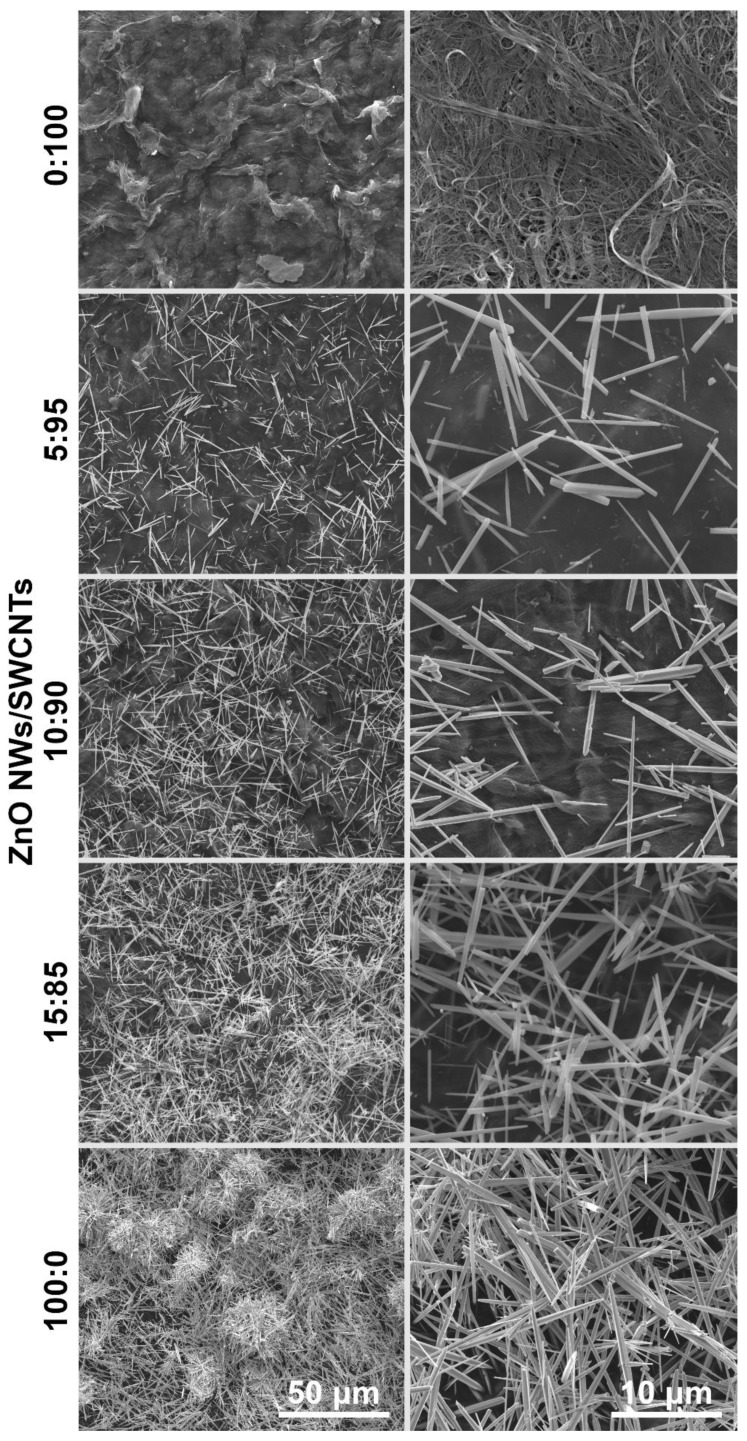
SEM micrographs of an SWCNT film, SWCNT films filled with ZnO NWs—5%/10%/15% by weight, and ZnO NWs.

Subsequently, the microstructure of SWCNTs, ZnO NWs, and their composites were studied by SEM (Figure 3). The starting SWCNT films were isotropic because filtration does not promote any degree of alignment, unless conducted under special conditions [34,35]. Bundles resulting from numerous van der Waals interactions between constituting CNTs can be clearly discerned. The amount of amorphous carbon, which typically contaminates CNT materials, was minimal. 

Furthermore, the synthesized ZnO NWs were pure and of appreciable aspect ratio. In this case, as well, no obvious signs of impurities could be noted, resonating well with the results of the XRD analysis described above, which illustrated that the NW material is pristine.

Lastly, three types of composites were visualized, which differed in the amount of ZnO NWs introduced to the SWCNT films (5 wt%, 10 wt%, and 15 wt%with respect to SWCNTs). ZnO NWs were homogeneously distributed within the SWCNT matrix. In addition, the gradual increase in the amount of ZnO NW filler is evident in the provided micrographs. It is important to mention that both the components were properly integrated, which is confirmed by HR SEM (Figure S1). Individual SWCNTs and their bundles are wrapped around the ZnO NWs, which should ensure adequate charge propagation. Lastly, similar to the starting SWCNT film, the composites are also porous, judging by the considerable content of cavities filled with air under ambient conditions.

**Figure 4:**
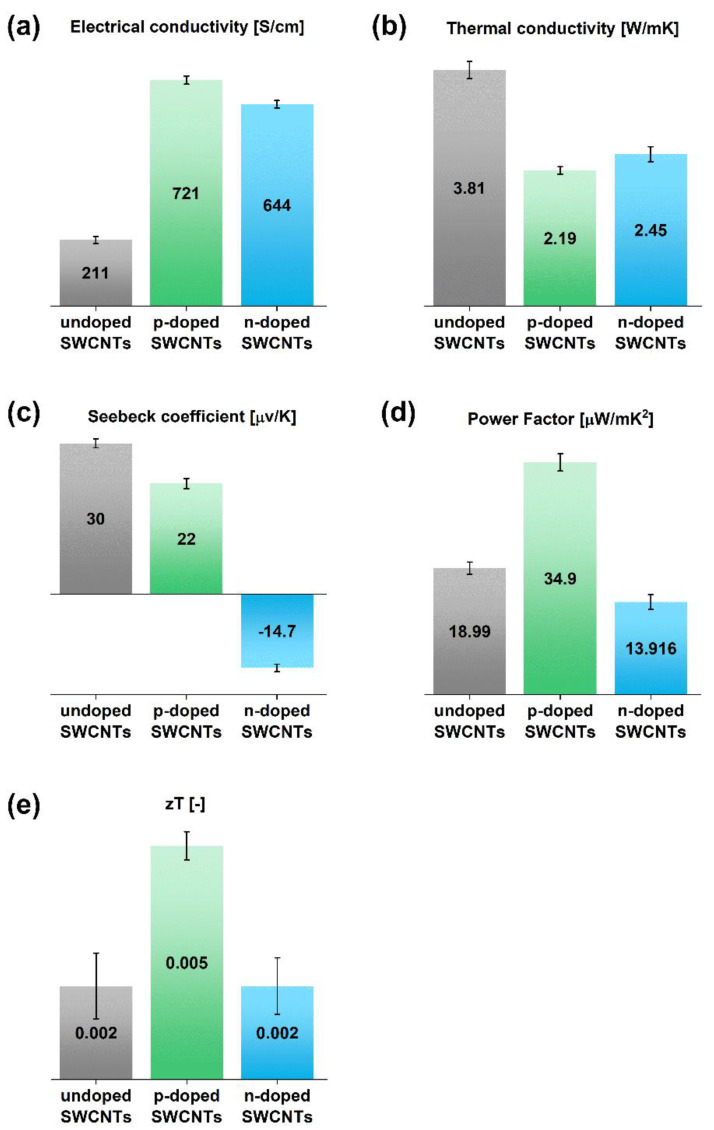
The impact of p- and n-doping of SWCNT films on the values of (**a**) electrical conductivity, (**b**) thermal conductivity, (**c**) Seebeck coefficient, (**d**) Power Factor, and (**e**) Figure of Merit.

Having defined the composition of the materials, characterization of the electrical, thermal and thermoelectric properties was conducted. A thermogenerator requires at least one pair of p- and n-doped thermoelements to operate. As-made SWCNT films are of p-type character due to the O_2_ doping effect [36]. However, oxygen is not a promising dopant for this application, as it may easily desorb at high temperatures. Therefore, it is necessary to add a chemical compound stable at high temperature, with as low vapor pressure as possible, capable of extracting electrons from the C(sp^2^) lattice of SWCNTs at elevated temperature. It was discovered that 1,2,3-triazole is a suitable dopant as it is electron-poor and its boiling point exceeds 200 °C [37] (Figure 4). Importantly, the addition of this chemical compound increased the capabilities of the SWCNTs to transport charge. While the starting SWCNT films had an electrical conductivity of 211 ± 11 S/cm, the value increased to 721 ± 13 S/cm upon doping with triazole, which was more than a three-fold improvement. Simultaneously, the thermal conductivity of the SWCNT ensemble decreased from 3.81 ± 0.14 W/m∙K to 2.19 ± 0.06 W/m∙K. It is beneficial, as minimization of thermal transport makes the temperature gradient necessary for the conversion of heat into electricity more stable.

Meanwhile, the Seebeck coefficient decreased from 30 ± 0.90 µV/K to 22 ± 1.01 µV/K. Optimizing the parameters of materials for thermoelectrics is challenging as they are interdependent [15]. Thus, an increase in electrical conductivity is often accompanied by the deterioration of the Seebeck coefficient, which is what we observed. Measures that can quantify the net effect of these changes are Power Factor (disregarding the impact of thermal conductivity) and Figure of Merit zT (including this factor in the consideration). The results indicate that despite the decrease in the Seebeck coefficient, p-doping of SWCNTs almost doubled the value of the PF (34.9 ± 1.3 µW/m∙K^2^ vs. 18.99 ± 0.9 µW/m∙K^2^). Furthermore, because of the desired decrease in thermal conductivity, the beneficial effect of triazole was even more pronounced in the case of the Figure of Merit, which increased from 0.002 to 0.005.

Finding an appropriate n-dopant for SWCNT-based thermogenerators is even more demanding, as it has to overcome the doping effect of oxygen present in the ambient. Moreover, it should be stable at high temperatures and, ideally, be unreactive toward the p-dopant, which may be emitted from the thermoelements, thereby decreasing the overall performance of the module. That is why commonly selected p-dopants, such as nitric [38] or sulfuric [39] acids, are not always applicable, if one considers the utilization of amines as n-dopants, as it may lead to an acid–base reaction. With this in mind, we decided to employ polyethyleneimine (PEI), which cannot react with triazole. Its addition caused a three-fold increase in electrical conductivity, with respect to the undoped SWCNT film, reaching 644 ± 12 S/cm. Furthermore, the thermal conductivity of the n-doped SWCNT film was 2.45 ± 0.12 W/m∙K, which was similar to when the nanocarbon was doped with a triazole. However, even though the sign of the Seebeck coefficient was changed, which indicated successful n-doping, the magnitude of this parameter was unsatisfactory i.e., −14.7 ± 0.75 µV/K. A high value of the Seebeck coefficient is necessary to reach appreciable thermoelectric performance, as in both Power Factor and the Figure of Merit, its effect is quadratic. This explains why, despite the increase in electrical conductivity, the Power Factor actually decreased to 13.92 ± 1.10 µW/m∙K^2^, compared with the starting material. In the case of the Figure of Merit, this problem was somewhat alleviated by the favorable decrease in the value of thermal conductivity. Nonetheless, the Figure of Merit remained at precisely the same level i.e., 0.002. This displeasing outcome motivated us to search for another material, the addition of which would lead to a higher n-doping performance.

**Figure 5:**
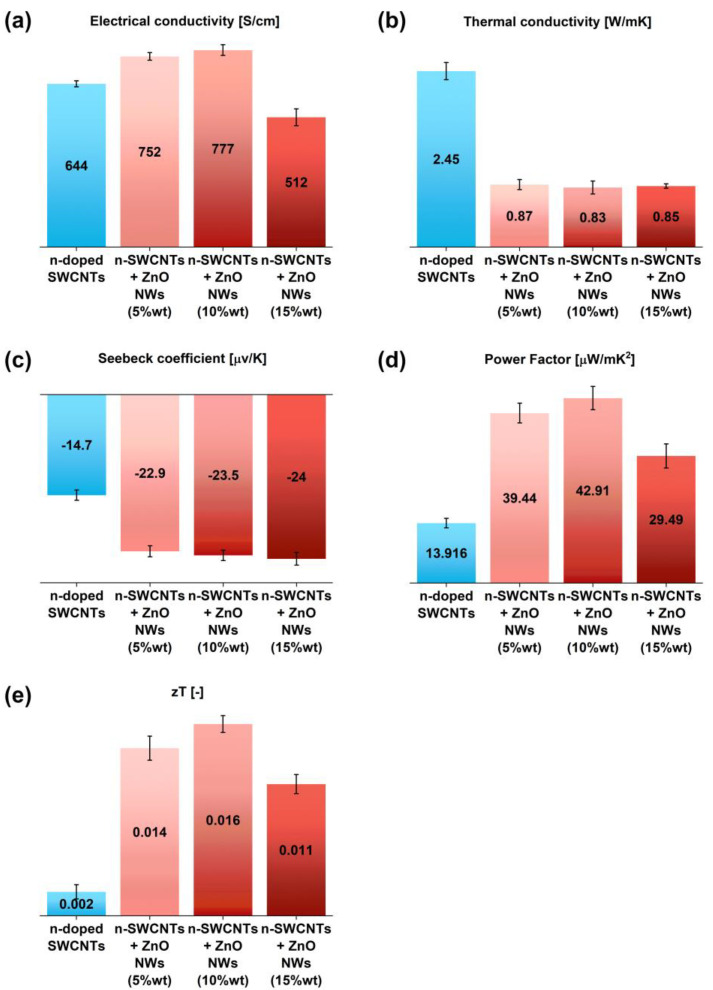
The influence of the incorporation of ZnO NWs (5 wt%, 10 wt%, and 15 wt% with respect to SWCNTs) into n-doped SWCNT films on the values of (**a**) electrical conductivity, (**b**) thermal conductivity, (**c**) Seebeck coefficient, (**d**) Power Factor, and (**e**) Figure of Merit.

Because of the previously reported n-type behavior of ZnO NWs [40,41], ZnO NWs were included in the n-doped SWCNT thermoelements (Figure 5). The addition was shown to improve the electrical conductivity of the network, up to the content of 10 wt% (777 ± 12 S/cm), after which it decreased with 15 wt% ZnO NWs (512 ± 10 S/cm). Moreover, a tangible decrease in thermal conductivity was also observed. This parameter was beneficially reduced by a factor of three, eventually reaching 0.83 W/m∙K. Apparently, the presence of ZnO NWs in the structure disturbs the heat transport between the individual SWCNTs. 

The biggest benefit of interfacing SWCNTs with ZnO NWs was the increase in the absolute values of the Seebeck coefficient. A gradual improvement in the thermopower was observed with the increasing ZnO NW amount. At 15 wt% of ZnO NWs, the Seebeck coefficient reached values as low as −24 ± 0.93 µV/K. Overall, when all three parameters (electrical conductivity, thermal conductivity, and Seebeck coefficient) are considered, the best net effect was obtained in the case of 10 wt% ZnO NWs in the material. The Power Factor increased from 18.99 ± 0.9 µW/m∙K^2^ to 42.91 ± 2.7 µW/m∙K^2^, which is an improvement of over 100%. Although the sample containing 15 wt% ZnO NWs exhibited the highest absolute value of the Seebeck coefficient, its influence was minimized by lower electrical conductivity with respect to the starting material. Lastly, because of the beneficial decrease in thermal conductivity for ZnO NW-enriched n-doped SWCNT thermoelements, the advantage coming from the implementation of ZnO NWs is even more evident, when looking from the Figure of Merit perspective. In this case, one can see an eight-fold improvement, as the value of zT increased from 0.002 to 0.016.

**Figure 6:**
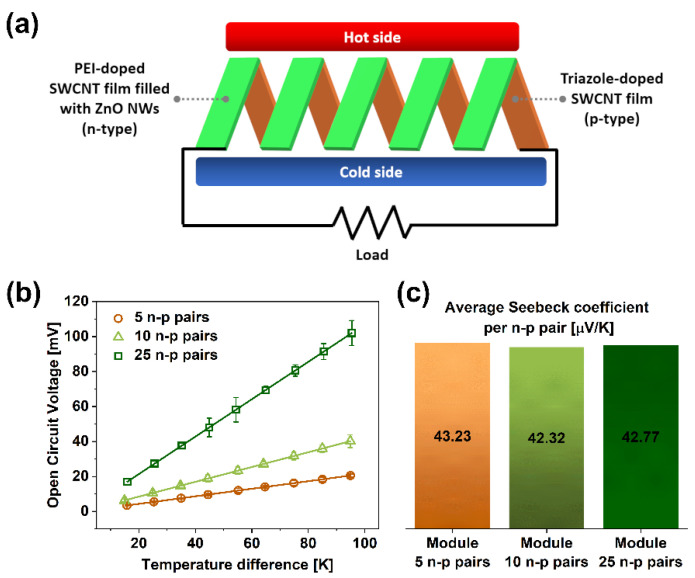
(**a**) Schematic representation of the evaluated module made from SWCNTs, ZnO NWs, and PEI/triazole as n- and p-dopants, respectively, (**b**) the magnitude of generated Open Circuit Voltage as a function of the temperature difference and the number of employed n–p pairs, and (**c**) average Seebeck coefficient per n–p pair determined from the slopes of the curves presented in panel (**b**).

Finally, the utility of the developed SWCNT-based thermoelements (n-type), containing PEI and ZnO NWs, as well as (p-type) employing triazole was demonstrated. Thermoelectric modules were constructed from 5, 10, and 25 n–p pairs made from the materials mentioned above (Figure 6). Then, the modules were subjected to various temperature gradients (Figure 6a), and the Open Circuit Voltage was measured (Figure 6b). The amount of electrical energy generated from waste heat was proportional to the magnitude of the temperature difference or the number of n–p pairs. Electric potential as large as 102 ± 7.14 mV was established for the biggest module at 95 °C difference. 

The results also suggest that the proposed solution is readily scalable (Figure 6c). Average Seebeck coefficients (determined from the slopes of the linear fits shown in Figure 6b) show virtually no changes, regardless of the number of employed n–p pairs. Consequently, it should be possible to obtain much more thermopower by creating modules containing many more n–p pairs. Alternatively, several modules could be connected in parallel or in series to increase the magnitude of the produced current or electric potential, respectively.

## 4. Conclusions

The study illustrates the versatile nature of SWCNTs, the properties of which can be readily adapted for an envisioned application, by mixing with appropriate chemical compounds or other components. Due to their amphoteric character, it was possible to obtain both p- and n-doped thermoelements by using triazole and polyethyleneimine, respectively. While the performance of the former hybrid was acceptable, it was necessary to include additional filler, in the case of the latter, to reach appreciable characteristics. Through the application of ZnO NWs, it was possible to increase the values of Power Factor and the Figure of Merit several times. A fully functioning prototype, capable of transforming waste heat into electricity, made from such elements, was made and tested. It was shown that such a module can generate a considerable amount of thermopower. Importantly, its operational parameters were found to be scalable, so it should be possible to increase its size to generate even more electrical energy.

Because of numerous types of nanomaterials, which fully cover the spectrum of potential properties, the number of possible combinations between them is virtually limitless. The birth of other promising types of nanomaterials, in recent years, such as transition metal dichalcogenides or MXenes, provides plenty of opportunities for further explorations into how the hybridization of several types of materials and nanomaterials may enable the tailoring of properties for particular applications.

## Figures and Tables

**Table 1:** Composition of the mixtures used to form SWCNT films in this study.

Sample	SWCNTs [mg]	PEI [M]	Triazole [M]	ZnO [mg]	Solvent Mixture [mL]
Pure SWCNT film	150.0	-	-	-	80
n-doped SWCNT film	150.0	0.1	-	-	80
n-doped SWCNT film filled with ZnO NWs (5 wt%)	142.5	0.1	-	7.5	80
n-doped SWCNT film filled with ZnO NWs (10 wt%)	135.0	0.1	-	15.0	80
n-doped SWCNT film filled with ZnO NWs (15 wt%)	127.5	0.1	-	22.5	80
p-doped SWCNT film	150.0	-	0.1	-	80

## Data Availability

Data from this study are available from the corresponding author upon a reasonable request.

## Supplementary Materials

The following supporting information can be downloaded at: https://www.mdpi.com/article/10.3390/ma15051924/s1, Figure S1: SEM micrograph of an SWCNT film filled with 15 wt% ZnO NWs by weight.
