# Supplementary material for: Carbon Nanotube-Based Thermoelectric Modules Enhanced by ZnO Nanowires
Source: Materials (Basel). 2022 Mar 4;15(5):1924. doi: 10.3390/ma15051924 (PMC8911611; doi:10.3390/ma15051924)
Supplement: Supplementary file 1 [file materials-15-01924-s001.zip › materials-1609829-supplementary.pdf]

*materials*

Communication

# Carbon Nanotube-Based Thermoelectric Modules Enhanced by ZnO Nanowires

**Patrycja Taborowska [1], Tomasz Wasiak [1], Mika Sahlman [2], Mari Lundström [2] and Dawid Janas [1,*]**

[1] Department of Organic Chemistry, Bioorganic Chemistry and Biotechnology, Silesian University of Technology, B. Krzywoustego 4, 44-100 Gliwice, Poland; patrycja.taborowska@polsl.pl (P.T.); tomasz.wasiak@polsl.pl (T.W.)
[2] Hydrometallurgy and Corrosion, Department of Chemical and Metallurgical Engineering (CMET), School of Chemical Engineering, Aalto University, P.O. Box 16200, FI-00076 Aalto, Finland; mika.sahlman@aalto.fi (M.S.); mari.lundstrom@aalto.fi (M.L.)
* Correspondence: dawid.janas@polsl.pl; Tel.: +48-32-237-10-82

## 1. HR SEM imaging of SWCNT-ZnO NW hybrids

The micrograph shown below illustrates the microstructure of an SWCNT film containing 15 wt% ZnO NWs. SWCNTs and SWCNT bundles are wound around individual ZnO NWs.

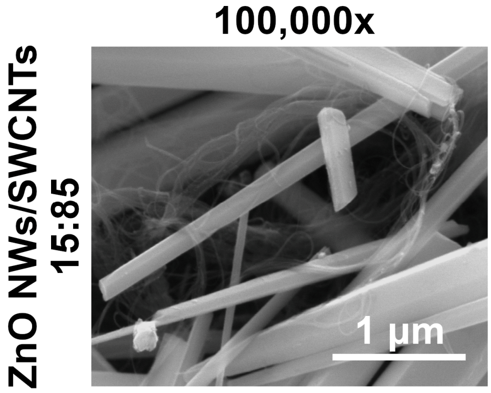


**Figure S1.** SEM micrograph of an SWCNT film filled with 15 wt% ZnO NWs by weight.

**Citation:** Taborowska, P.; Wasiak, T.; Sahlman, M.; Lundström, M.; Janas, D. Carbon Nanotube-Based Thermoelectric Modules Enhanced by Zno Nanowires. *Materials* **2022**, *15*, 1924. https://doi.org/10.3390/ma15051924

Academic Editor: Nuno Ferreira

Received: 8 February 2022
Accepted: 3 March 2022
Published: 4 March 2022

**Publisher's Note:** MDPI stays neutral with regard to jurisdictional claims in published maps and institutional affiliations.

**Copyright:** © 2022 by the authors. Licensee MDPI, Basel, Switzerland. This article is an open access article distributed under the terms and conditions of the Creative Commons Attribution (CC BY) license (https://creativecommons.org/licenses/by/4.0/).
